# A Study on the Preparation of a Vulcanizing Mixture and Its Application in Natural Rubber Latex

**DOI:** 10.3390/polym16091256

**Published:** 2024-04-30

**Authors:** Haobin Fang, Yingping He, Yulan Li, Jie Du

**Affiliations:** School of Materials Science and Engineering, Hainan University, Haikou 570228, China; 21220856000011@hainanu.edu.cn (H.F.); 990344@hainanu.edu.cn (Y.H.); 20170481310149@hainanu.edu.cn (Y.L.)

**Keywords:** natural latex, vulcanization mixture, mechanical properties, process optimization

## Abstract

The traditional preparation process of natural rubber latex requires tedious treatment of a variety of rubber additives. In this paper, a new process of wet mixed grinding was used to prepare a reinforced vulcanization mixture and a rapid vulcanization effect. The effect of different amounts of vulcanization mixtures on the mechanical properties of natural latex film was studied, and the pre-vulcanization process of latex and the vulcanization process of film were optimized. The results showed that with the increase in the amount of vulcanization mixture, the tensile strength increased from 5.96 MPa to 29.28 MPa, and the tear strength increased from 7.59 kN/m to 52.81 kN/m. When the vulcanization temperature of the latex film is heated from 80 °C to 100 °C, the vulcanization time is shortened by 5~6 times. The new vulcanization mixture prepared in this work has the characteristics of simple production and fast vulcanization speed, which provides a new solution for the development of the latex product industry.

## 1. Introduction

Natural rubber is a green renewable natural resource. Since its discovery, after years of technological development, it has been made into a variety of products and integrated into people’s daily lives [1,2]. With the excellent comprehensive performance of natural rubber, it has been made into aviation tires, latex pillows, toy balloons, condoms, catheters, and other products [3,4,5,6].

The polyisoprene molecular chain, the main component of natural rubber, exists in natural rubber latex particles. The balance of latex particles is broken, which makes the rubber molecular chain release, and then the molecular chain accumulates to form natural rubber raw rubber compound. A large number of natural rubber molecular chains are connected to each other to form a huge rubber molecular network, forming a natural rubber material with high strength and high elongation [7,8,9]. Sulfur is a common rubber molecular chain vulcanizing agent that is used to achieve the purpose of crosslinking. Relying on the advantage of cheap prices, sulfur is also used in the processing and production of synthetic rubber. Under the sulfur-based vulcanized rubber system, a series of additives, such as accelerators and activators, have been studied and applied [10,11,12]. At the same time, many researchers have added different reinforcing materials or modified materials to natural rubber to improve the performance of natural rubber [13,14,15,16].

However, there are still some problems in the development of natural rubber materials. For example, the properties of unprocessed rubber are very weak, the additives added are not easy to disperse in natural rubber latex, and the traditional processing technology is cumbersome [17,18,19]. In view of the above problems, in recent years, relevant researchers have developed different kinds of vulcanization accelerators and reinforcing agents, developed new tools for pretreatment of additives, and developed new equipment to improve the production process [20,21,22,23,24].

With the advent of the low-carbon era, green and environmentally friendly materials are more and more popular. It is still a research hotspot in recent years to continuously improve the performance of green natural rubber materials and expand their application scope [25,26,27,28]. For natural rubber composites, the added reinforcing agent and accelerator can be uniformly dispersed in the system to ensure sufficient crosslinking reaction inside. At present, the liquid phase mixing method is a convenient and quick method. In the liquid state, the natural rubber latex is mixed with the required dispersant, and then the natural rubber composite material with excellent performance is prepared by vulcanization treatment [29].

This paper hopes to develop a vulcanization mixture for natural rubber latex, which can solve the problem of the tedious grinding process of rubber additives in the production process of natural rubber latex ingredients. The method of mixing and grinding rubber auxiliaries step by step is put forward to prepare the vulcanization mixture, which has the characteristics of simple production and rapid vulcanization, and the suitable processing conditions are determined by comparing the dosage and optimizing the production process. This will provide a new idea for the preparation of super strong natural rubber composite materials with natural latex film. Based on a similar processing process, the application of a vulcanization mixture in synthetic latex also has potential research value.

## 2. Materials and Methods

### 2.1. Materials

Concentrated latex, a mass fraction of 61.5%, was provided by the Hainan Jinlian Rubber Processing Branch (Danzhou, China). Sulfur, industrial grade, was purchased from Langli Chemical Co., Ltd. (Shanghai, China). Bis(ethylphenylcarbamodithioato-S,S′)-, (T-4)-Zinc (Accelerator PX), 98% purity, was purchased from the Shanghai Myrill Biochemical Technology Co., Ltd. (Shanghai, China). Zinc carbonate, 68.5% purity, was purchased from the Suzhou Ketong Biomedical Technology Co., Ltd. (Suzhou, China). Silica, industrial grade, was purchased from the Shuanglong Chemical Co., Ltd. (Shanghai, China). 2,6-Ditert-butyl-4-methyl phenol(Antioxidant 264), chemical pure, was purchased from the Shanghai Bohr Chemical Reagent Co., Ltd. (Shanghai, China). 2-Mercaptobenzothiazole(Accelerator M), purity 98%, was purchased from the Shanghai Jizhi Biochemical Technology Co., Ltd. (Shanghai, China). Sodium salt of polynaphthalene sulphonic acid (Dispersing agent NNO), 95% purity, was purchased from the Shanghai Bohr Chemical Reagent Co., Ltd. (Shanghai, China). Potassium hydroxide, analytically pure, was purchased from the Guangzhou Chemical Reagent Factory (Guangzhou, China). Aqua ammonia, concentration 25–28%, was purchased from the Shanghai McLean Biochemical Technology Co., Ltd. (Shanghai, China). Pure water was made by the laboratory.

### 2.2. Preparation of Samples

#### 2.2.1. Preparation of the Vulcanization Mixture

The KNB-S-1L horizontal sand mill from the Dongguan Kangbo Intelligent Equipment Co., Ltd. (Dongguan, China) was used to carry out step by step wet mixed grinding of various rubber additives. The selection of rubber additives and the experimental formula are from the previous experiments, which are detailed in Table S1. The experimental formula is shown in Table 1. At the initial moment, only sulfur and silica were added, followed by antioxidant 264; then, two accelerators were added, and finally, zinc carbonate was added.

#### 2.2.2. Preparation of Film under Different Dosages of the Vulcanization Mixture

According to the experimental formula in Table 2, different parts of the vulcanization mixture were mixed with natural latex, and the latex was pre-vulcanized. Ammonia was added to maintain the stability of the latex during the pre-vulcanization of the latex. The pre-vulcanization conditions were set to 60 °C, 1.5 h; after that, the film was spread on the glass plate. After the film was dried for 1 to 2 days, the film was vulcanized. The vulcanization conditions were set to 80 °C and 2 h. From the experimental results of vulcanization mixture, the appropriate dosage was determined through comprehensive calculation, and the subsequent experiment was continued. The calculation process was shown in Table S2.

#### 2.2.3. Preparation of Latex with Different Pre-Vulcanization Times

The latex was vulcanized by water bath heating. The water bath heating temperature was set to 60 °C, and the heating was started from room temperature; the set temperature can be reached in about thirty minutes. The latex was slowly stirred during the pre-vulcanization process to make it evenly heated. According to the experimental design in Table 3, regular sampling was carried out, and corresponding tests were carried out. The PVT0 sample was not pre-vulcanized, the PVT1 sample had just reached the pre-vulcanization setting temperature, and the subsequent sample pre-vulcanization time was separated by 30 min.

#### 2.2.4. Preparation of Films under Different Vulcanization Conditions

According to the experimental design in Table 4, the oven temperature was designed to be 80 °C, 90 °C, and 100 °C, respectively, and different time intervals were designed. The oven reaches the set temperature and starts the timing experiment. The arrival time quickly removes the film and stands for 24 h, and then the mechanical properties are tested.

### 2.3. Characterization Methods

The particle size test of rubber additives was carried out using an HL2020-c laser particle size analyzer from the Haixinrui Technology Co., Ltd. (Beijing, China). Under the wet test standard, the shading degree (optical concentration) was about 15%.

The tensile strength and tear strength of the vulcanized film were determined by an AI-7000-SU2 tensile machine from the High-Speed Rail Technology Co., Ltd. (Taiwan, China). With reference to the national standards [30,31], the vulcanized film was cut into a dumbbell shape and a right angle shape with a specific knife, and the tensile machine rate was set to 500 mm/min.

The functional relationship between the amount of vulcanization mixture and the mechanical properties was established. The amount of vulcanization mixture was X value, and the mechanical properties were Y value. The polynomial curve fitting was carried out using the simple fit plug-in from OriginPro 2022. At the same time, the confidence band of the fitting curve was marked according to 95% confidence.

After the tensile strength test, the film was completely broken. A Phenom ProX scanning electron microscope (SEM) from the PhenOM-World BV company (Eindhoven, The Netherlands) was used to observe the microstructure of the film. The surface of the film was treated with gold spray before observation.

The vulcanization characteristics of the film were tested using the MDR-2000E rotor-free vulcanization instrument from the Wuxi Liyuan Electronic Chemical Equipment Co., Ltd. (Wuxi, China). With reference to the national standard [32], the experimental temperature was set at 100 °C, and the time was 30 min.

Latex viscosity was tested using the VISCOBALL type falling ball viscimeter from the Spinning Jilaibo Technology Co., Ltd. (Beijing, China). At room temperature (25 °C), the time it took for the test ball to roll a fixed length in the measuring tube equipped with latex was counted, and the dynamic viscosity was calculated according to Formula (1).

H, dynamic viscosity of latex; k, test ball constant, 0.09 in this experiment; ρ_1_, test ball density, 2.20 in this experiment; and ρ_2_, latex density, 0.94 in this experiment.

H = k × (ρ_1_ − ρ_2_) × t (1)

The thermal stability of the latex was tested by a rotary viscometer. The mechanical stability of the latex was tested by a Dunlop–Klaxon latex mechanical stability tester. Toluene solution was used as a solvent to test the swelling rate of the film size.

## 3. Results

### 3.1. Preparation of the Vulcanization Mixture

In our previous experiment of grinding rubber additives alone, we observed some phenomena, as shown in Figure S1. The process of grinding alone is more complicated, so we control the feeding sequence according to the nature of different rubber auxiliaries. Sulfur and silica texture is relatively hard and difficult to grind. Antioxidant 264 is a large crystalline particle. The use of preliminary mortar grinding makes the accelerator powder particles easy to grind. Zinc carbonate grinding makes it excessively easy to produce bubbles that effect the quality of the vulcanization mixture.

**Figure 1:**
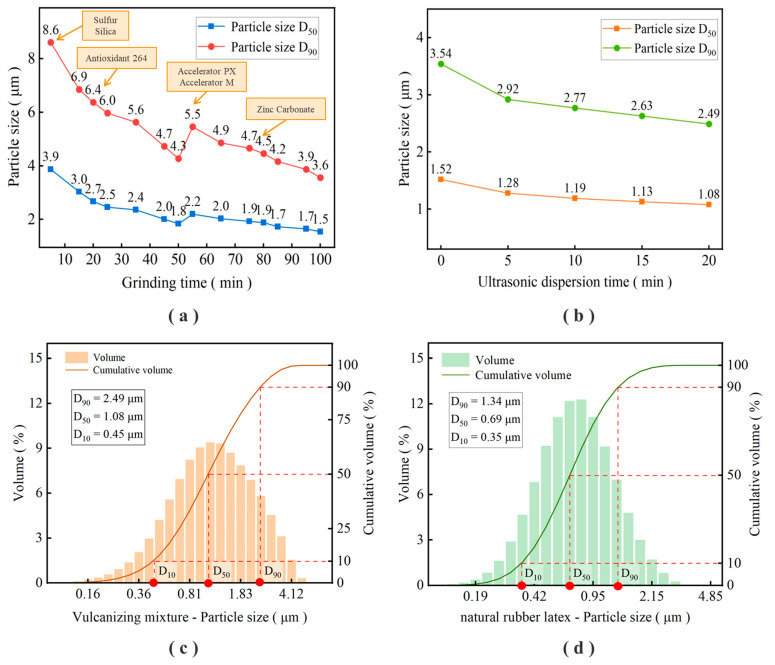
Refining process and particle size distribution curve of the vulcanized mixture: (**a**) particle size change during grinding of the vulcanization mixture; (**b**) particle size change during ultrasonic treatment of the vulcanization mixture; (**c**) particle size distribution of the final vulcanization mixture (**d**) particle size distribution of natural latex.

In order to achieve better grinding results, only sulfur and silica are added at 0 min, antioxidant 264 min after 20 min, accelerator PX and accelerator MBT after 55 min, zinc carbonate 20 min from the end, and dispersant NNO is added in stages during grinding. After the vulcanization mixture is ground, ultrasonic dispersion treatment is continued to achieve further refinement of the particle size. The particle size changes during the test, and the test results are shown in Figure 1.

As can be seen from the results in Figure 1a, the particle size of the rubber auxiliary decreases continuously with the grinding process. This is because in the grinding tank, the zirconia grinding beads collide with the rubber auxiliaries, making the rubber auxiliaries constantly broken and forming finer particles. The particle size increases in the middle process due to the addition of two non-ground accelerators, and then the particle size of the mixture decreases with the extension of the grinding time. According to the results in Figure 1b, ultrasonic dispersion treatment can further reduce the particle size of rubber auxiliaries. Finally, the particle size of the mixed vulcanization mixture was reduced to 2.49 μm D_90_ and 1.08 μm D_50_.

It can be seen from the distribution of two particle sizes in Figure 1c,d that the particle size of the cured vulcanization mixture is close to that of the natural latex. The fine treatment of rubber additives is the key to solving its dispersion in latex, and good dispersion can promote a better reaction between rubber additives and latex.

### 3.2. Influence and Analysis of Curing Mixture Dosage on the Mechanical Properties of the Film

**Figure 2:**
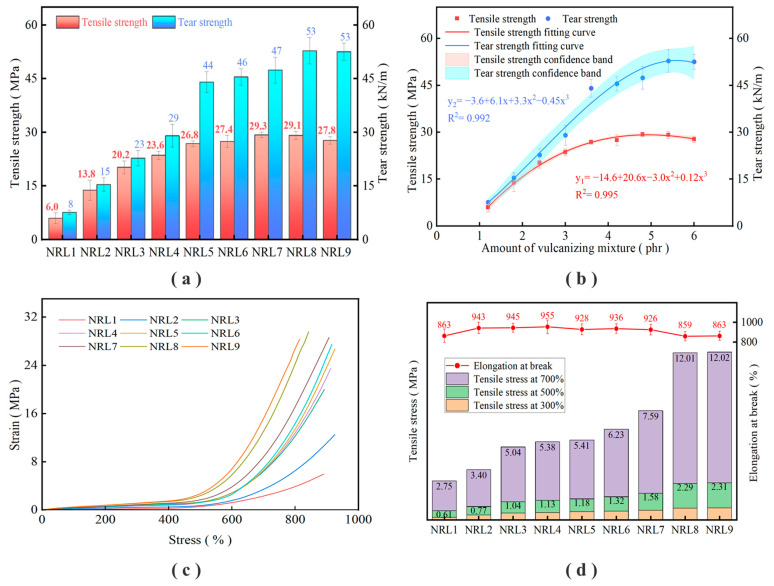
Mechanical properties of the film under different dosages of the vulcanization mixture: (**a**) mechanical properties of the film; (**b**) fitting curve of the mechanical properties; (**c**) stress–strain curve; (**d**) tensile stress and elongation at break.

**Figure 3:**
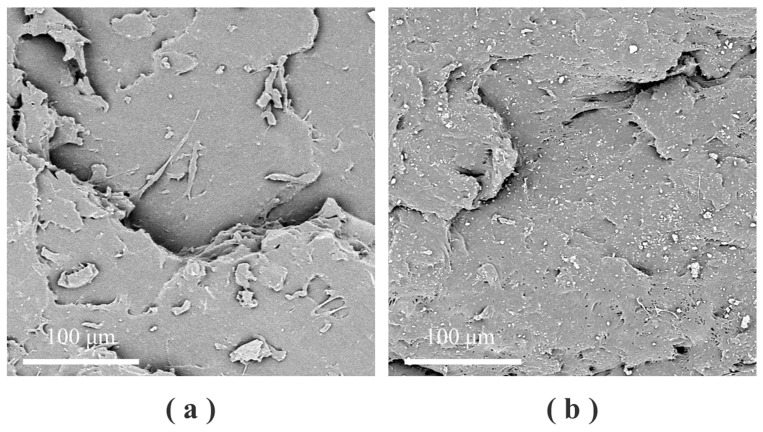
Scanning electron microscopy (SEM) image of the sample: (**a**) NRL1; (**b**) NRL9.

The mechanical properties of vulcanized rubber films with different amounts of the vulcanized mixture were tested. The results are shown in Table 5 and Figure 2. Polynomial curve fitting was carried out with the amount of the vulcanization package as X value and the mechanical properties as Y value. At the same time, the confidence band of the fitting curve was marked according to 95% confidence, and the results are shown in Figure 2b. Scanning electron microscopy (SEM) was used to observe the microstructure of the film section, and the results are shown in Figure 3.

In Table 5 and Figure 2a, the different amounts of the curing mixture had different effects on the mechanical properties of the vulcanized film. The mechanical properties increased rapidly at a small amount, and then gradually stabilized. The tensile strength increased from the initial 5.96 MPa to 29.28 MPa, and the tear strength increased from the initial 7.59 kN/m to 52.81 kN/m.

In Figure 2b, the polynomial curve fitting was carried out with the amount of vulcanization mixture as the X value and the mechanical properties as the Y value. The relationship between the tensile strength and the amount of vulcanization mixture is y_1_ = −14.6 + 20.6x − 3.0x^2^ + 0.12x^3^, R^2^ = 0.995; the relationship between the tear strength and the amount of vulcanization mixture is y_2_ = −3.6 + 6.1x + 3.3x^2^ − 0.45x^3^, R^2^ = 0.992. From the confidence band of the shadow part, it can be seen that the curve fitting effect of the tensile strength is better, the R^2^ value is larger, and the result is more credible. The curve fitting effect of tear strength is weaker, which is also consistent with the test process. In the test process of tear strength, the same sample test error is larger.

It can be seen in Figure 2c that the stress–strain curve moves upward with the increase in the amount of the vulcanization mixture. At the same time, under the condition of low elongation (0~400%), the difference between the stress–strain curves is not large, and the difference between the curves is mainly reflected in the stage of high elongation (500~900%). It can be seen in Figure 2d that with the increase in the amount of vulcanization mixture, the tensile stress also showed an upward trend, and the elongation at break increased first and then decreased.

In Figure 3, the cross-section of the film is an uneven layered structure, and the white spot-like substance is the added rubber auxiliary. A variety of rubber auxiliaries were refined in the early stage to prepare the vulcanization mixture, and it was introduced into the rubber system, still maintaining fine particles. Figure 3a shows the NRL1 sample, in which the amount of the vulcanized mixture is less, and it is easily dispersed in the latex film. Figure 3b shows the NRL9 sample. At this time, the amount of the vulcanization mixture is high, but it can still maintain a good dispersion effect in the latex film, which proves that the earlier refinement treatment has achieved its purpose. Rubber auxiliaries have a good dispersion effect in latex film, which will lay a foundation for their full play in latex.

The vulcanization mixture contains various rubber additives, which cause the vulcanization reaction of natural rubber latex film. The addition of the vulcanization mixture will promote the formation of more crosslinking structures in natural rubber latex film. Natural latex contains a small number of proteins, lipids, etc. These original components can promote a small number of crosslinking reactions in the latex film. At this time, the film structure is not strong enough and does not have the ability to resist external forces, and the film performance is weak. With the increase in the amount of the vulcanization mixture, more crosslinking structures were formed between the rubber molecular chains, which greatly improved the mechanical properties of the film [33,34,35,36,37,38].

When the curing mixture increases at a small amount, a partial crosslinked structure is formed inside the film. At this time, the film performance is weak and is in an unstable stage of mechanical performance growth. The tensile stress of the latex film is low, and the stress–strain curve of the latex film increases slowly. With the increase in the amount of the curing mixture, more crosslinking structures were generated between the molecular chains, and the film with good tensile properties was formed. At this time, the tensile strength gradually reached the peak value, the tensile stress gradually increased, the stress–strain curve increased obviously, and the elongation at break gradually stabilized. After exceeding the optimal value, the amount of the vulcanization mixture continued to increase, and a closer crosslinked structure was formed in the film, making it difficult for the film to be pulled to a larger length and showing that the tear strength no longer increased, and the tensile strength and elongation at the break began to decrease gradually.

### 3.3. Effects and Analysis of Different Pre-Vulcanization Times on the Properties of Latex and Film

#### 3.3.1. Effect and Analysis of Different Pre-Vulcanization Times on the Properties of Latex and Unvulcanized Film

**Figure 4:**
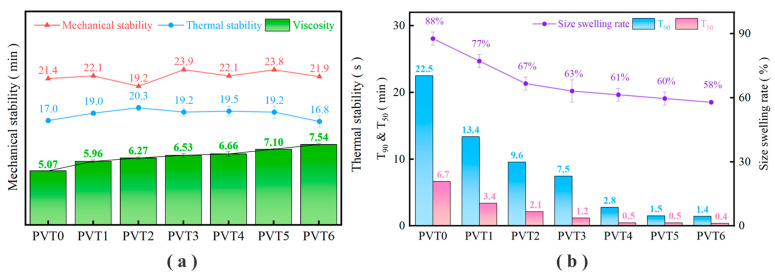
Properties of latex film under different pre-vulcanization times: (**a**) thermal stability, mechanical stability, and the viscosity of latex; (**b**) vulcanization characteristics and the dimensional swelling ratio of the film.

The experiment mainly tested the thermal stability, mechanical stability, and viscosity of the latex under different pre-vulcanization times. The test results are shown in Figure 4a. After the latex film was dried at room temperature, the swelling rate of the film size and the vulcanization characteristics of the film were tested. The test results are shown in Table 6 and Figure 4b.

It can be seen in Figure 4a that with the pre-vulcanization of the latex, the mechanical properties of the latex remained at about 20 min, the thermal stability of the latex increased first and then decreased, and the viscosity of the latex increased slowly. In Figure 4b, it can be seen that the size swelling rate of the film prepared by latex after different pre-vulcanization operations decreases with the increase in pre-vulcanization times and then tends to be stable, and the vulcanization characteristic values T_90_ and T_50_ all show a decreasing trend.

As an important latex production step, pre-vulcanization has a great influence on the properties of latex. During the pre-vulcanization process, the latex absorbs heat, which increases the activity of the latex particles through heat transfer, changes the original equilibrium system of the latex particles, and may change the morphology of some latex particles [39,40]. At the same time, the vulcanization mixture may be partially adsorbed on the surface of the latex particles in the latex, which promotes the crosslinking of some latex particles and the appearance of fine particles in the latex [41,42,43,44].

The change in latex properties is mainly due to the rupture of some latexes, and more latexes rupture with the increase in pre-vulcanization time [45,46,47]. Mechanical stability represents the ability of latex to resist mechanical damage. Through high-speed stirring, a large number of latex particles are broken, and finally, coagulates appear in the latex. The thermal stability shows that under heat treatment, the latex particles react with the zinc ammonia complex particles to form a complex structure, which makes the latex become a rubber clot; the increase in the viscosity of the latex is mainly due to the change in the appearance of more latex particles, the enhancement of the interaction between each other, and the obstruction of the liquid flow of the latex, which is manifested as the increase in the viscosity of the latex.

The change in film properties may be due to the fact that the rubber molecular chain contacts with the substances in the vulcanization mixture in advance and reacts to form a crosslinked structure. With the increase in pre-vulcanization time, more latex particles are activated, and it is easier to react in the subsequent vulcanization process; thus, the film properties are improved [48,49]. The unvulcanized film was prepared from latex with different pre-vulcanization times, which showed different vulcanization characteristic values and size swelling rates. The increase in the pre-vulcanization time promotes the advanced vulcanization reaction of more latex particles, and the unvulcanized film has a greater degree of vulcanization and crosslinking. Therefore, it is shown that the vulcanization characteristic values T_90_ and T_50_ are reduced and the dimensional swelling rate is reduced.

#### 3.3.2. Effect and Analysis of Different Pre-Vulcanization Times on the Mechanical Properties of the Vulcanized Film

**Figure 5:**
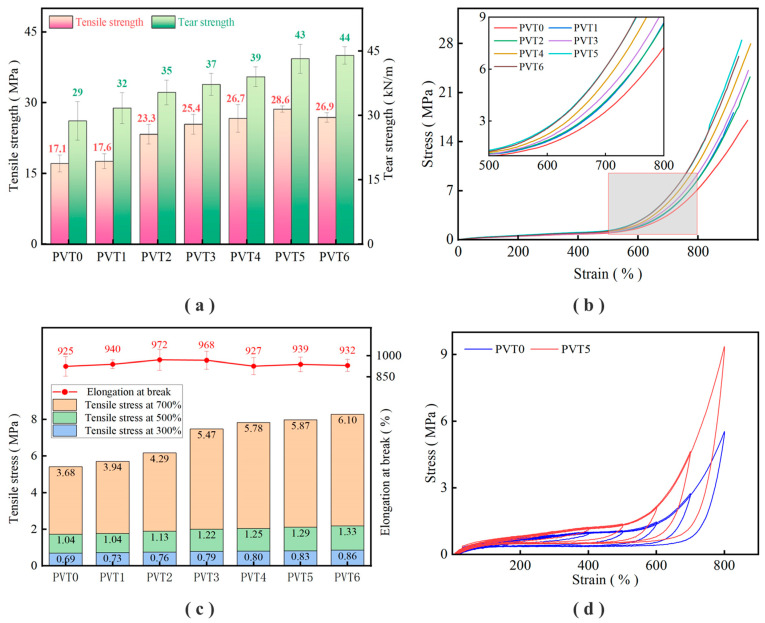
Test results of mechanical properties of the vulcanized film under different pre-vulcanization times: (**a**) tensile strength and tear strength; (**b**) stress–strain curve and local amplification diagram; (**c**) tensile strength and elongation at break; (**d**) cyclic tensile curve.

After different pre-vulcanization times, the latex was dried, the film was vulcanized to prepare the vulcanized film, and the mechanical properties were tested. The test results are shown in Table 7 and Figure 5.

The test results in Table 5 show that different pre-vulcanization times have different effects on the mechanical properties of the obtained vulcanized film. The tensile strength increased from the initial 17.11 MPa to 28.62 MPa, and the tear strength increased from the initial 28.74 kN/m to 44.01 kN/m.

It can be seen in Figure 5a that the tensile strength and tear strength increase with the increase in the pre-vulcanization time. The tensile strength decreases after reaching the maximum value, and the tear strength gradually increases with the increase in the pre-vulcanization time and then tends to be stable. It can be seen in Figure 5b that the stress–strain curve moves upward, indicating that the tensile strength of the film is improved.

It can be seen in Figure 5c that the tensile strength of the film was improved with the extension of the pre-vulcanization time, and the elongation at break was basically maintained at about 900%. From the results in Figure 5d, the length of the sample increases continuously after stretched and gradually returns to the original state after releasing the tension. Compared with the two samples, PVT0 and PVT5, after pre-vulcanization, there will be greater stress when the film is stretched to the same length, and this phenomenon is more obvious at higher deformation lengths.

In the pre-vulcanization process, the vulcanization reaction occurs in advance between the rubber molecular chain and the sulfur, which can make it easier to achieve a state of excellent performance and show higher mechanical properties [7,46,50].

With the increase in pre-vulcanization time, the latex gradually reaches a stable state, and the performance of the film will also have a better state. At this time, the tensile strength and tearing strength gradually reach the ideal level, and the elongation at the break is gradually stable. After continuing to increase the pre-vulcanization time, the degree of the pre-vulcanization of latex exceeds the most appropriate state, and excessive vulcanization may occur for the vulcanization film. The advantage of the rubber molecular chain being pre-vulcanized gradually disappeared. As the tensile strength decreases, the tearing strength, the elongation at the break, and the strength at fixed elongation no longer increase.

### 3.4. Test and Analysis of the Mechanical Properties of Rubber Film under Different Curing Times

**Figure 6:**
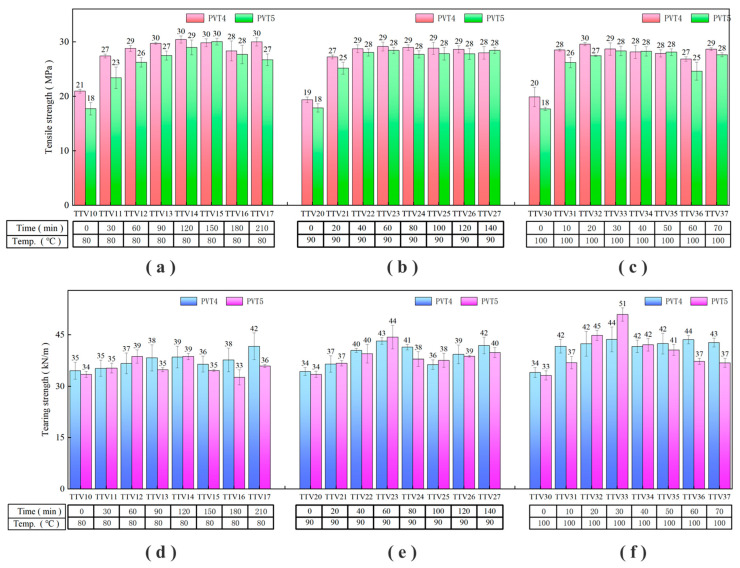
Test results of the mechanical properties of rubber film under different vulcanization conditions: (**a**–**c**) the tensile strength results of the film at 80 °C, 90 °C, and 100 °C, respectively; (**d**–**f**) the results of the tear strength of the film at 80 °C, 90 °C, and 100 °C, respectively.

By considering the properties of the latex colloid and comparing the mechanical properties of the film, the appropriate pre-vulcanization time of the latex was selected for the vulcanization mixture. The experimental conditions of samples PVT4 and PVT5 were selected, and the pre-vulcanization time was 90 min and 120 min, respectively. On the basis of determining the amount of the vulcanization mixture, the optimization of vulcanization conditions was continued under the determined pre-vulcanization conditions. The effects of different vulcanization temperatures and vulcanization times on latex and film were studied. The experimental conditions were designed as shown in Table 4, mainly considering the tensile strength and tear strength of the film, as shown in Figure 6. Detailed test data were shown in Table S3.

It can be seen in Figure 6 that after vulcanization at different temperatures, the mechanical properties of the film will increase with the increase in vulcanization time, and then will tend to be gentle. At 80 °C, the film sample basically peaked at the vulcanization time of 120~150 min; at 90 °C, the film sample basically peaked at the vulcanization time of 40~60 min; and at 100 °C, the film samples basically peaked at the vulcanization time of 20~30 min. Comparing the two samples, PVT4 and PVT5, the peak value of the mechanical properties of PVT4 will be slightly higher than the peak value of PVT5, and the peak value is slightly ahead of the time.

The Improvement of the mechanical properties of the latex film is mainly due to the sulfur and accelerators inside the latex film, which promote the vulcanization reaction of the rubber molecular chain and the formation of disulfide bonds between the rubber molecular chains. The molecular chains are entangled with each other to form a network structure, which promotes the increase in the mechanical properties of the film [51,52,53,54].

Therefore, the curing time was shortened by 5~6 times when the curing temperature was increased from 80 °C to 100 °C. The pre-vulcanization time of the PVT4 sample was shorter than that of the PVT5 sample, but the performance of the final film reached a higher level. It may be that under this experimental condition, a short time of pre-vulcanization can save the consumption of the vulcanization mixture in the latex, and it is enough to complete the dispersion of the vulcanization mixture in the latex, so the film with better performance is finally obtained.

## 4. Conclusions

(1) The rubber additives were subjected to step-by-step mixing, grinding, and ultrasonic dispersion treatment. The particle size D_90_ of the prepared mixed vulcanization mixture was reduced to 2.49 μm, and D_50_ was reduced to 1.08 μm. The particle size is equivalent to the size of latex particles, and the fine treatment of rubber additives is realized.
(2) The effects of different amounts of the vulcanization mixture on the mechanical properties of latex film were compared. With the increase in the amount of the vulcanization mixture, the mechanical properties of latex film were greatly improved. The tensile strength of the latex film increased from 5.96 MPa to 29.28 MPa, and the tear strength increased from 7.59 kN/m to 52.81 kN/m.
(3) With the prolongation of the pre-vulcanization time, the viscosity of latex gradually increased, the vulcanization characteristic values of unvulcanized film T_90_ and T_50_ decreased, and the mechanical properties of vulcanized film improved. The appropriate pre-vulcanization time was determined to be 90 min and 120 min, respectively.
(4) Increasing the vulcanization temperature can greatly reduce the vulcanization time of the film, and after the vulcanization temperature rises from 80 °C to 100 °C, the vulcanization time can be shortened by five to six times. Vulcanized film with good mechanical properties can be obtained when the vulcanization temperature is 100 °C and the vulcanization time is 20~30 min.

## Figures and Tables

**Table 1:** The dry base ratio of rubber auxiliaries in the vulcanization mixture.

Material	Sulfur	Silica	Antioxidant 264	Accelerator PX	Accelerator M	Zinc Carbonate	Dispersing Agent NNO
Proportion (phr)	1.20	1.00	0.50	0.60	0.30	0.50	0.10

**Table 2:** Experimental formulations under different dosages of the vulcanization mixture.

Sample	NRL1	NRL2	NRL3	NRL4	NRL5	NRL6	NRL7	NRL8	NRL9
Natural latex (phr)	100	100	100	100	100	100	100	100	100
Vulcanization mixture (phr)	1.2	1.8	2.4	3.0	3.6	4.2	4.8	5.4	6.0
Aqua ammonia (mL)	0.5	0.5	0.5	0.5	0.5	0.5	0.5	0.5	0.5

**Table 3:** Experimental design of different pre-vulcanization times of latex.

Sample	PVT0	PVT1	PVT2	PVT3	PVT4	PVT5	PVT6
Pre-vulcanization time (min)	/	0	30	60	90	120	150

**Table 4:** Experimental design of different vulcanization temperatures and different vulcanization times of rubber film.

Sample		1	2	3	4	5	6	7	8
Sample group 1	Temperature (80 °C)	TTV10	TTV11	TTV12	TTV13	TTV14	TTV15	TTV16	TTV17
	Time (min)	0	30	60	90	120	150	180	210
Sample group 2	Temperature (90 °C)	TTV20	TTV21	TTV22	TTV23	TTV24	TTV25	TTV26	TTV27
	Time (min)	0	20	40	60	80	100	120	140
Sample group 3	Temperature (100 °C)	TTV30	TTV31	TTV32	TTV33	TTV34	TTV35	TTV36	TTV37
	Time (min)	0	10	20	30	40	50	60	70

**Table 5:** Mechanical properties test results of rubber film under different dosages of the vulcanization mixture.

Sample	300% Tensile Strength (MPa)	500% Tensile Strength (MPa)	700% Tensile Strength (MPa)	Elongation at Break (%)	Tensile Strength (MPa)	Tear Strength (kN/m)
NRL1	0.25	0.61	2.75	863	5.96 ± 1.49	7.59 ± 0.61
NRL2	0.47	0.77	3.40	943	13.82 ± 2.80	15.42 ± 1.87
NRL3	0.64	1.04	5.04	944	20.22 ± 1.79	22.76 ± 2.14
NRL4	0.69	1.13	5.38	955	23.58 ± 1.13	29.05 ± 3.21
NRL5	0.76	1.18	5.41	928	26.82 ± 0.79	44.06 ± 2.93
NRL6	0.82	1.32	6.23	936	27.43 ± 1.72	45.51 ± 2.32
NRL7	0.89	1.58	7.59	925	29.28 ± 0.78	47.40 ± 3.64
NRL8	1.09	2.29	12.01	859	29.13 ± 1.13	52.81 ± 3.68
NRL9	1.12	2.31	12.02	863	27.75 ± 1.07	52.55 ± 2.40

**Table 6:** Vulcanization characteristic parameters of the unvulcanized film.

Sample	T_10_ (min)	T_50_ (min)	T_90_ (min)	M_H_ (dN·m)	M_L_ (dN·m)	M_H_-M_L_ (dN·m)
PVT0	2.22	6.65	22.50	1.54	0.75	0.79
PVT1	1.70	3.40	13.35	1.76	0.65	1.11
PVT2	0.77	2.12	9.57	1.79	0.75	1.04
PVT3	0.40	1.17	7.47	1.49	0.62	0.87
PVT4	0.23	0.45	2.77	0.74	0.54	0.20
PVT5	0.25	0.47	1.43	1.12	0.87	0.25
PVT6	0.22	0.40	1.52	1.10	0.73	0.37

**Table 7:** Mechanical properties test results of the vulcanized film under different pre-vulcanization times.

Sample	300% Tensile Strength MPa	500% Tensile Strength MPa	700% Tensile Strength MPa	Elongation at Break %	Tensile Strength MPa	Tear Strength kN/m
PVT0	0.67	0.97	3.56	965	17.11 ± 1.79	28.74 ± 4.50
PVT1	0.76	1.09	4.09	918	17.82 ± 1.60	31.73 ± 3.61
PVT2	0.76	1.12	4.16	973	23.30 ± 2.07	35.38 ± 2.87
PVT3	0.72	1.07	4.63	968	25.42 ± 2.11	37.26 ± 2.55
PVT4	0.80	1.19	5.32	976	26.66 ± 2.93	39.04 ± 2.28
PVT5	0.85	1.32	6.11	945	28.62 ± 0.67	43.23 ± 3.39
PVT6	0.80	1.25	6.10	936	26.86 ± 0.99	44.01 ± 2.00

## Data Availability

The original contributions presented in the study are included in the article/Supplementary Material, further inquiries can be directed to the corresponding author.

## Supplementary Materials

The following supporting information can be downloaded at https://www.mdpi.com/article/10.3390/polym16091256/s1, Figure S1. The change in particle size D_90_ of different kinds of rubber auxiliaries in separate grinding processes. Table S1. The dry base ratio of rubber auxiliaries in the vulcanization mixture. Table S2. The contribution of the unit sulfide mixture and the calculation results of the comprehensive score. Table S3. Test results of mechanical properties of vulcanized rubber film under different vulcanization conditions.
