# Supplementary material for: A Study on the Preparation of a Vulcanizing Mixture and Its Application in Natural Rubber Latex
Source: Polymers (Basel). 2024 Apr 30;16(9):1256. doi: 10.3390/polym16091256 (PMC11085568; doi:10.3390/polym16091256)
Supplement: Supplementary file 1 [file polymers-16-01256-s001.zip › polymers-2993530-supplementary.pdf]

Study on the preparation of vulcanizing mixture and its application in natural rubber latex

Haobin Fang, Yingping He, Yulan Li, Jie Du*

School of Materials Science and Engineering, Hainan University, Haikou 570228, China

1. Different kinds of rubber auxiliaries are ground separately under different grinding conditions

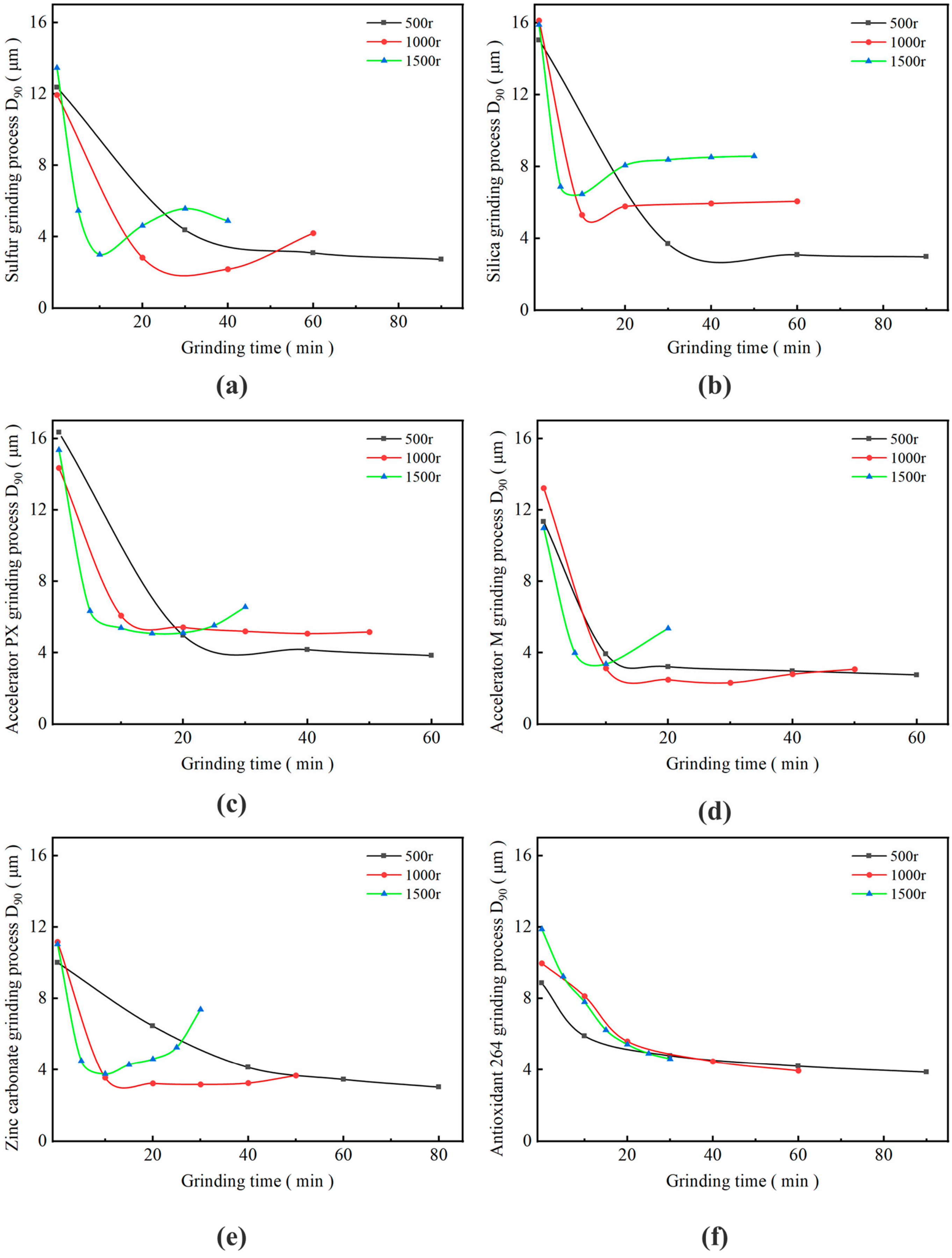

Figure S1 The change of particle size D_{90} of different kinds of rubber auxiliaries in separate grinding process

In our previous research work, another study on the grinding and refining treatment of rubber auxiliaries explored the particle size changes during separate grinding of different rubber auxiliaries. It was found that the sulfur and the silica, need more grinding time than other rubber auxiliaries to achieve the same refinement effect under the same grinding process.

Probably because sulfur and silica are harder to grind; Antioxidant 264 is a large crystalline particle, which needs to be initially ground with a mortar before formal wet grinding; The accelerant is powder particle, which is easy to grind, and the excessive grinding of zinc carbonate is easy to produce bubbles and affect the quality of vulcanization mixture.

Separate grinding process is relatively complicated, so we according to different properties of rubber additives, by controlling the sequence, achieve the goal of the mixed grinding.After a large number of experimental attempts, we obtained the final grinding feeding sequence.

Sulfur and silicon dioxide were first added to the grinding tank, and after 20 minutes, peated-ground antioxidant 264 was added after 55 minutes, accelerator PX and accelerator M were added, and zinc carbonate was added 20 minutes from the end. Dispersant NNO was added in stages during the grinding process.

2. Vulcanization mixture formula

Table S1. Dry base ratio of rubber auxiliaries in vulcanization mixture

Material	Sulfur	Silica	Antioxidant 264	Accelerator PX	Accelerator M	Zinc carbonate	Dispersing agent NNO
proportion (phr)	1.20	1.00	0.50	0.60	0.30	0.50	0.10

The vulcanization mixture formulation came from another study on formulation design.

The sulfur-based vulcanization system was selected, and two different kinds of vulcanization accelerators were selected, namely, dithiocarbamate accelerator-accelerator PX and thiazole accelerator-accelerator M. The choice of two accelerators is expected to obtain the complementary and mutual activation between the accelerators, to achieve higher vulcanization speed, to achieve higher application value.

Silica was selected as the reinforcing agent of latex film. In the previous study, it was verified that silica had certain reinforcing effect.

3. Comprehensive consideration of the amount of curing mixture and determination of the final amount

In order to comprehensively consider the contribution of the vulcanized mixture to the mechanical properties, and determine an appropriate dosage for the subsequent experiments.

The unit contribution of the vulcanized mixture was calculated as shown in Formula A1. The contribution of phr per unit vulcanization mixture to tensile strength and tear strength test results were calculated respectively, denoted as unit contribution 1 and unit contribution 2.In order to eliminate the influence of the measurement unit in the unit contribution, the membership degree was again calculated separately, as shown in Formula A2. Membership of unit contribution 1 and unit contribution 2 are calculated respectively, denoted as membership 1 and membership 2.Comprehensive scores were calculated by considering the mechanical properties and assigning weights to membership respectively, as shown in Formula A3.Membership 1 and 2 were assigned weights of 50% and 50%, respectively.All the calculated results are shown in Table A2.

$$\text{Unit contribution} = \text{Mechanical properties Properties/curing mix amount} \quad (A1)$$

$$\text{Membership} = (\text{index value x- index minimum})/(\text{index maximum - index minimum}) \quad (A2)$$

$$\text{Comprehensive score} = \text{Membership 1*50\%+ Membership 2*50\%} \quad (A3)$$

Table S2 The contribution of unit sulfide mixture and the calculation results of comprehensive score

	**1**	**2**	**3**	**4**	**5**	**6**	**7**	**8**	**9**
Sample name	NRL1	NRL2	NRL3	NRL4	NRL5	NRL6	NRL7	NRL8	NRL9
Tensile strength (MPa)	5.96	13.82	20.22	23.58	26.82	27.43	29.28	29.13	27.75
Tearing strength (kN/m)	7.59	15.42	22.76	29.05	44.06	45.51	47.40	52.81	52.55
Amount of vulcanization mixture (phr)	1.2	1.8	2.4	3.0	3.6	4.2	4.8	5.4	6.0
Unit contribution 1 (MPa/phr)	4.97	7.68	8.42	7.86	7.45	6.53	6.10	5.39	4.63
Unit contribution 2 (kN/(m·phr))	6.32	8.57	9.48	9.68	12.24	10.84	9.88	9.78	8.76
Membership 1	0.09	0.80	1.00	0.85	0.74	0.50	0.39	0.20	0.00
Membership 2	0.00	0.38	0.53	0.57	1.00	0.76	0.60	0.58	0.41
Comprehensive score	0.04	0.59	0.77	0.71	0.87	0.63	0.49	0.39	0.21

In this experimental study, when the amount of vulcanizing mixture was 2.4 phr, the contribution of vulcanizing mixture to tensile strength reached the maximum. When the amount of vulcanizing mixture was 3.6 phr, the contribution of vulcanizing mixture to tear strength reached the maximum. The comprehensive score represents the contribution of the vulcanization mixture to the comprehensive mechanical properties of the latex film. By comparing the comprehensive scores, the amount of 3.6 phr was finally determined. The subsequent pre-vulcanization experiment and vulcanization experiment were carried out according to this formula.

4.Vulcanization experiment mechanical properties test results

In the experiment of pre-vulcanization of latex, two batches of latex were obtained, which were PVT4 and PVT5. Two batches of latex films were obtained by spreading the latex. Three experimental groups were set up for the latex film, and different time intervals were set at 80 °C, 90 °C and 100 °C, respectively, to obtain samples with different vulcanization conditions. After the mechanical properties test, the results are shown in Table S3.

Table S3 Test results of mechanical properties of vulcanized rubber film under different vulcanization conditions

**Sample group**	**Sample**	**300 % tensile strength (MPa)**	**500% tensile strength (MPa)**	**700% tensile strength (MPa)**	**Elongation at break (%)**	**tensile strength (MPa)**	**tear strength (kN/m)**
PVT4	TTV10	0.69	1.54	7.35	888	20.95±0.34	34.57±2.46
	TTV11	0.78	1.47	7.39	934	27.40±0.37	35.28±2.34
	TTV12	0.83	1.45	7.35	929	28.79±0.54	36.66±3.00
	TTV13	0.80	1.27	5.77	950	29.72±0.18	38.31±3.79
	TTV14	0.84	1.26	5.21	999	30.44±0.65	38.54±3.14
	TTV15	0.81	1.23	5.17	996	29.82±0.73	36.48±2.30
	TTV16	0.82	1.27	5.34	983	28.33±1.82	37.71±3.43
	TTV17	0.82	1.31	6.09	971	29.99±0.76	41.68±3.91
	TTV20	0.66	1.34	5.71	929	19.37±0.52	34.35±1.18
	TTV21	0.79	1.36	6.71	939	27.22±0.34	36.51±2.37
	TTV22	0.87	1.54	8.00	923	28.73±0.73	40.44±0.6
	TTV23	0.92	1.57	7.99	919	29.13±0.82	43.21±0.97
	TTV24	0.86	1.34	6.08	956	28.95±0.52	41.41±0.80

	TTV25	0.85	1.29	5.48	969	28.83±1.10	36.35±1.39
	TTV26	0.81	1.25	5.42	947	28.62±0.65	39.31±2.69
	TTV27	0.84	1.28	5.42	948	27.98±1.15	41.88±2.42
	TTV30	0.69	1.58	7.24	879	19.88±1.77	34.07±1.47
	TTV31	0.89	1.74	9.22	897	28.50±0.20	41.67±1.99
	TTV32	0.89	1.54	8.02	922	29.57±0.30	42.40±3.64
	TTV33	0.94	1.70	8.93	899	28.67±1.13	43.65±3.64
	TTV34	0.82	1.25	4.98	993	28.14±1.21	41.62±1.81
	TTV35	0.81	1.28	5.62	961	27.87±0.66	42.45±3.03
	TTV36	0.80	1.23	5.10	968	26.84±0.41	43.60±1.24
	TTV37	0.83	1.32	6.11	950	28.66±0.27	42.75±1.27
PVT5	TTV10	0.62	1.28	5.63	872	17.71±1.13	33.5±0.89
	TTV11	0.76	1.37	6.86	866	23.41±1.99	35.34±1.40
	TTV12	0.79	1.30	6.27	943	26.24±0.87	38.68±1.93
	TTV13	0.79	1.33	6.62	940	27.49±0.83	34.87±0.58
	TTV14	0.79	1.22	5.58	970	28.98±1.36	38.69±0.88
	TTV15	0.85	1.28	5.56	980	30.03±0.63	34.65±0.29
	TTV16	0.76	1.13	4.58	1000	27.69±1.69	32.63±2.18
	TTV17	0.81	1.28	6.28	938	26.71±1.08	35.94±0.43
	TTV20	0.64	1.49	6.56	875	17.88±0.77	33.52±0.89
	TTV21	0.81	1.47	7.28	897	25.17±1.10	36.76±0.76
	TTV22	0.83	1.49	7.83	919	28.06±0.66	39.51±2.69
	TTV23	0.86	1.43	7.20	929	28.43±0.59	44.34±3.48
	TTV24	0.80	1.23	4.98	965	27.70±0.67	37.96±2.15
	TTV25	0.78	1.23	5.26	977	27.82±1.16	37.60±2.05
	TTV26	0.79	1.24	5.38	947	27.80±1.01	38.73±0.25
	TTV27	0.85	1.27	5.12	975	28.42±0.57	39.85±1.46
	TTV30	0.68	1.33	5.63	923	17.71±0.30	33.20±1.33
	TTV31	0.80	1.27	5.95	952	26.20±0.95	36.92±1.80
	TTV32	0.87	1.62	8.95	862	27.45±0.11	44.85±1.48
	TTV33	0.83	1.28	5.73	960	28.34±0.86	50.95±1.94
	TTV34	0.86	1.33	5.99	947	28.26±0.85	42.09±1.83
	TTV35	0.83	1.26	5.04	982	28.11±0.62	40.60±1.58
	TTV36	0.85	1.43	7.22	888	24.62±1.66	37.29±0.87
	TTV37	0.85	1.26	5.35	957	27.64±0.37	36.87±1.28
